# Anterior segment parameters in patients with coronavirus disease

**DOI:** 10.5935/0004-2749.20210087

**Published:** 2021

**Authors:** Kemal Örnek, Emine Temel, Özkan Kocamış, Nazife Aşıkgarip, Lokman Hızmalı

**Affiliations:** 1 Department of Opthalmology, Kırşehir Ahi Evran University School of Medicine, Kırşehir, Turkey; 2 Department of Opthalmology, Kırşehir Ahi Evran Training and Research Hospital, Kırşehir, Turkey; 3 Department of Infectious Disease and Clinical Microbiology, Kırşehir Ahi Evran University School of Medicine, Kırşehir, Turkey

Dear Editor,

Since first identified in December 2019 at Wuhan, China, the coronavirus disease (COVID-19) has become a major public health challenge wordlwide^(1)^. As of September 4, 2020, more than 26 million COVID-19 cases, including 869,600 deaths, were reported in 216 countries^(2)^.

The ophthalmological alterations caused by COVID-19 are mainly limited to external ocular diseases such as conjunctivitis^(3,4)^. Therefore, in patients with the infection, we measured anterior segment (AS) parameters, which may show subtle changes in the acute phase of the infection and serve as a tool for monitoring the long-term changes after recovery from COVID-19.

This prospective study included patients who were hospitalized for confirmed COVID-19 infection (Group 1) and healthy subjects (Group 2). The right eye of each participant was examined. All the patients in group 1 were positive for COVID-19 in real-time reverse transcriptase-polymerase chain reaction tests using nasopharyngeal swabs. The study was conducted with the approval of our institutional review board and adhered to the principles of the Declaration of Helsinki. Informed consent was obtained from all the participants.

Central corneal thickness, aqueous depth, anterior chamber depth, lens thickness, and white-to-white corneal diameter were measured with a Lenstar LS 900 device (Hagg-Streit AG, Koeniz, Switzerland). All the between-group comparisons were statistically analyzed using SPSS 11.5 (SPSS Inc., Chicago, IL, USA).

Group 1 consisted of 32 COVID-19 patients (32 eyes), whereas group 2 was comprised of 34 healthy subjects (34 eyes). The mean age was 35.9 ± 21.6 years (range, 8-87 years) in group 1 and 37.2 ± 14.9 years (range, 10-63 years) in group 2. The female-to-male ratio was 14:18 in group 1 and 20:14 in group 2. No significant difference was found between the groups in terms of age and sex (p=0.6 and p=0.4, respectively).

None of the patients had coexisting ocular symptoms. No abnormalities of the cornea, anterior chamber, or posterior segment were found in the slit-lamp examination. When compared, the values of the quantitative AS parameters were not significantly different between the groups (Table 1).

**Table 1:** Comparison of the quantitative anterior segment parameters between the groups

	Group 1 (n=32)	Group 2 (n=34)	P value
CCT (µm) Mean ± SD (Range)	525.8 ± 31.3 (454-599)	528.2 ± 29.1 (465-576)	0.751
AD (mm) Mean ± SD (Range)	3.04 ± 0.38 (2.49-4.59)	2.86 ± 0.41 (1.75 ± 3.58)	0.08
ACD (mm) Mean ± SD (Range)	3.56 ± 0.38 (3.02-5.11)	3.39 ± 0.41 (2.3-4.09)	0.09
LT (mm) Mean ± SD (Range)	3.92 ± 0.39 (3.15-4.67)	3.92 ± 0.45 (3.21-4.89)	0.95
WTW (mm) Mean ± SD (Range)	12.4 ± 0.68 (11.27-14.29)	12.02 ± 0.46 (11.25-13.4)	0.07

CCT= Central corneal thickness; SD= Standard deviation; AD= Aqueous depth; ACD= Anterior chamber depth; LT= Lens thickness; WTW= White-to-white corneal diameter.

COVID-19 infection causes an inflammatory response with the release of a large amount of proinflammatory cytokines. The host immune response to the virus is hyperactive, resulting in an excessive inflammatory reaction in the whole body. Anatomically, the cornea and anterior chamber structures use nutrients present in the aqueous humor, which is derived from plasma in the capillary network of the ciliary processes. Therefore, we hypothesized that aqueous proinflammatory cytokines derived from systemic circulation in patients with COVID-19 infection may be associated with changes in the corneal and AS parameters.

All available data on ocular COVID-19 manifestations are focused on the ocular surface and mostly derived from the external eye and conjunctival samples. Pirraglia et al. recently investigated the corneal sensitivity in COVID-19 patients and did not find any of the reductions known in herpes viruses^(5)^. This is the first study to quantitatively evaluate AS parameters in the eyes of patients with COVID-19 without manifestations of AS involvement.

In our study, the AS parameters in the COVID-19 patients did not seem to have significant changes as compared with those in the controls. The small sample size, lack of mild and severe patient groups, and inability to measure the anterior chamber inflammatory status are the limitations of the study. Future studies with longer follow-up periods are needed to show whether corneal and AS parameters are affected by COVID-19 and will help to understand the changes in COVID-19.
